# Fat accretion measurements strengthen the relationship between feed conversion efficiency and Nitrogen isotopic discrimination while rumen microbial genes contribute little

**DOI:** 10.1038/s41598-018-22103-4

**Published:** 2018-03-01

**Authors:** Sarah J. Meale, Marc D. Auffret, Mick Watson, Diego P. Morgavi, Gonzalo Cantalapiedra-Hijar, Carol-Anne Duthie, Rainer Roehe, Richard J. Dewhurst

**Affiliations:** 1 Université Clermont Auvergne, INRA, VetAgro Sup, UMR Herbivores, F-63122 Saint-Genès-Champanelle, France; 2 SRUC, West Mains Road, Edinburgh, EH9 3JG United Kingdom; 3 Division of Genetics and Genomics, The Roslin Institute and R(D)SVS, University of Edinburgh, Edinburgh, EH25 9RG United Kingdom; 4 Edinburgh Genomics, The Roslin Institute and R(D)SVS, University of Edinburgh, Edinburgh, EH25 9RG United Kingdom; 5 Present Address: School of Agriculture and Food Sciences, The University of Queensland, Gatton, QLD 4343 Australia

## Abstract

The use of biomarkers for feed conversion efficiency (FCE), such as Nitrogen isotopic discrimination (Δ^15^N), facilitates easier measurement and may be useful in breeding strategies. However, we need to better understand the relationship between FCE and Δ^15^N, particularly the effects of differences in the composition of liveweight gain and rumen N metabolism. Alongside measurements of FCE and Δ^15^N, we estimated changes in body composition and used dietary treatments with and without nitrates, and rumen metagenomics to explore these effects. Nitrate fed steers had reduced FCE and higher Δ^15^N in plasma compared to steers offered non-nitrate containing diets. The negative relationship between FCE and Δ^15^N was strengthened with the inclusion of fat depth change at the 3^rd^ lumbar vertebrae, but not with average daily gain. We identified 1,700 microbial genes with a relative abundance >0.01% of which, 26 were associated with Δ^15^N. These genes explained 69% of variation in Δ^15^N and showed clustering in two distinct functional networks. However, there was no clear relationship between their relative abundances and Δ^15^N, suggesting that rumen microbial genes contribute little to Δ^15^N. Conversely, we show that changes in the composition of gain (fat accretion) provide additional strength to the relationship between FCE and Δ^15^N.

## Introduction

Improving the efficiency of ruminants is an important goal due to the rising human population, growing competition for feed and food resources and increased concerns about environmental consequences of ruminant production. Feed efficiency is central to this issue, but can be difficult to measure, so there has been recent interest in potential biomarkers using samples that are easy to collect. One such biomarker is the phenomenon of Nitrogen isotopic discrimination (Δ^15^N), or the difference in natural ^15^N abundance (δ^15^N) between animal proteins and the diet. Variability in Δ^15^N has been linked to the efficiency of N metabolism^1,2^ and rumen microbial N metabolism^3^. At the mechanistic level, differences in the rates of biochemical reactions causing N isotopic discrimination contribute to the overall observed discrimination (Δ^15^N). Previous studies have reported negative relationships between FCE and Δ^15^N^4,5^, suggesting that the relationship with FCE may stem from its strong association with N intake and growth, thus reflecting the incorporation of N into tissue versus its excretion in urine^6^ – hence current interest in estimating the composition of gain.

Early work by Wattiaux and Reed^3^ indicated that rumen microbial metabolism can cause N isotopic discrimination, with effects particularly related to the incorporation and emission of ammonia into and out of microbial amino acids, respectively. Some previous studies suggested that rumen effects may be significant when there is relatively low variation in hepatic deamination and transamination^7^. Studies in other ecosystems such as soils^8^, oceans^9^ and in pure bacterial cultures^10^ have identified N isotopic discrimination associated with denitrification and it is possible that supplementation with dietary nitrate could enhance this pathway in ruminants^11^. Additionally, supplementation with nitrate linearly increases rumen ammonia concentrations, which is more energetically favourable than the reduction of carbon dioxide to methane^12,13^, theoretically increasing the availability of dietary energy. Yet, the effect of nitrate supplementation on N isotopic discrimination has not been studied and should be considered when determining its suitability as a biomarker for the feed efficiency phenotype. Furthermore, dietary lipids can have large effects on the number of rumen microbes^14^, which may influence the processes of denitrification and conversion of nitrate to ammonia, warranting investigation of its inclusion alone, and in combination with nitrate, on N isotopic discrimination. Overall, this study aimed to improve understanding of the relationship between FCE and Δ^15^N by (i) using ultrasound measurements to describe the composition of liveweight gain during a FCE test; and (ii) using dietary treatments and rumen metagenomics to explore the effects of rumen N metabolism across different diets.

## Materials and Methods

This experiment was conducted at Scotland’s Rural College (SRUC) Beef and Sheep Research Centre in Edinburgh in 2014. The experimental protocol was approved by SRUC’s Animal Welfare and Ethical Review Body, the Animal Experiments Committee and was conducted in accordance with the requirements of the UK Animals (Scientific Procedures) Act, 1986.

### Experimental design, animals and diets

Details of the experimental design, and growth and performance results have been reported previously^15^. Briefly, eighty 13–15 month old Aberdeen Angus (AAx) and Limousin (LIMx) sired steers, from an established rotational crossing study, were tested for feed efficiency. In a 2 × 4 factorial design (breed × diet), animals were offered a basal diet of 55:45 forage (grass and whole crop barley silages) to concentrate (DM basis), in which the main protein source (rapeseed meal), was replaced with 1) no additive (CONT; n = 20); 2) 128 g/kg DM maize dark grains containing ~12% lipids (LIPID; n = 20); 3) calcium nitrate at a maximum of 25 g/kg diet DM (NIT; n = 20; Calcinit, Yara, Oslo, Norway); or, 4) a combination of LIPID and NIT at the listed inclusion rates (COMB; n = 20). A full description of dietary ingredients and chemical composition is presented in Duthie *et al*.^15^. Steers in the NIT and COMB groups were progressively adapted to the calcium nitrate supplement over a 4 week period receiving 25, 50, 75 and 100% of the dietary nitrate each week. Treatments were balanced for sire within each breed and live-weight (BW) at the start of the experiment. Fresh water was provided for *ad libitum* intake, animals were bedded on sawdust and diets were offered using 32 electronic feeders (8 per pen, Insentec BV, Marknesse, The Netherlands) to record individual daily feed intakes.

### Feed efficiency test

Feed efficiency was estimated over a 56 d test period. Individual dry matter intake (DMI, kg/d) was recorded daily and BW was measured weekly, before fresh feed was offered, using a calibrated weigh scale. Hyslop *et al*.^16^ evaluated the precision of average daily gain (ADG) measurements over alternative test lengths and demonstrated that a 56 d measurement period, with weekly weighing is sufficient for characterising ADG of finishing beef cattle. For all steers, ultrasonic muscle and fat depth was obtained at the 10^th^ and 12^th^/13^th^ ribs, and at the 3^rd^ lumbar vertebrae at the start and end of the 56 d test using industry-standard equipment (Aloka 500, BCF Technology Ltd, Bellshill, UK). Images were analysed using Matrox Inspector 8 software (Matrox Video and Imaging Technology Europe Ltd., Middlesex, UK). Changes in body composition were calculated as the difference between the start and final depth (in mm). Following the feed efficiency test, 18 steers with extreme high or low FCE, balanced for breed type and diet were selected for further metagenomic analysis. Following the feed efficiency test, steers remained on the same experimental diets for a period of up to 14 weeks before slaughter, to allow measurement of methane emissions in all animals (once per animal in batches of six, as reported by Duthie *et al*.^15^). Animals were slaughtered in larger batches, comprising multiple measurement batches. Transportation to the abattoir took 1 hour and there was less than 3 hours between the animals having *ad libitum* access to feed and slaughter.

### Rumen sampling and analysis

Post-mortem digesta samples collected immediately after the rumen was opened to be drained (<45 min post-slaughter) in a commercial abattoir, were taken from the selected 18 steers, following our previous discovery that rumen samples from slaughter or live cattle showed high microbial community similarities^17^. Digesta was filtered through two layers of muslin cloth and samples of rumen liquid were transferred on dry ice then stored at −20 °C until analysis.

### DNA extractions

DNA was extracted from the rumen samples following the protocol described in Rooke *et al*.^18^. Briefly, the method involved a combination of repeated bead beating followed by column filtration based on the protocol of Yu and Morrison^19^.

### Metagenomic analysis

Illumina TruSeq libraries were prepared from genomic DNA and sequenced on Illumina HiSeq systems 4000 by Edinburgh Genomics. Further analyses of the data set followed the same procedure previously described in Wallace *et al*.^20^ and Roehe *et al*.^17^. Briefly, paired-end reads (2 × 100 bp) were generated, resulting in between 8.6 and 14.5 GB per sample (between 43.4 and 72.7 million paired reads). The microbial functional genes encoding for proteins and including the genes detailed in this study were identified using the KEGG genes database^21^. Parameters were adjusted such that all hits were reported that were equal in quality to the best hit for each genomic read. The read and best hits have to be more than 90% identical and have to be belonging to a single KEGG orthologue group to be kept in the data. If the best hits are spread over more than one KEGG orthologue group, the reads were disregarded. Read counts for KEGG orthologues were summed and normalised to the total number of hits. Then, annotated genes with a relative abundance of more than 0.01% were selected for further analysis. These data can be downloaded from the European Nucleotide Archive under accession PRJEB21624.

### Nitrogen isotopic discrimination of plasma proteins

Samples of feeds were taken weekly during the FCE test period and blood was sampled from the caudal vein (9 ml lithium heparin evacuated tubes; Greiner Bio One Ltd., Gloucestershire, UK) on the final day of the test period (d 56). Plasma was separated by centrifugation at 2 500 × *g* for approximately 15 min (Thermo Scientific, Heraeus Multifuge 3SR + centrifuge, Waltham, MA, USA) and stored at −20 °C until analysis. Weekly samples of each diet were dried at 60 °C and ground. Four milligram sub-samples of each diet, and plasma samples (20 μl) were prepared for ^15^N analysis by isotope ratio mass spectrometry (Iso-Analytical Ltd., Crewe, UK). Nitrogen isotopic discrimination (Δ^15^N) was calculated as the difference between the natural abundance of ^15^N in plasma proteins minus the natural abundance of ^15^N in the diet, such that Δ^15^N = δ^15^N_animal_ − δ^15^N_diet_. Results were expressed using the delta notation in parts per 1000 (‰) related to the international standard (air).

### Calculations and statistical analyses

Data from one steer from the 56 d test period was discarded as the steer was removed from the trial for health reasons unconnected to the diets and treatments imposed, leaving data from 79 steers available for analysis. Across all 79 steers, growth was modelled by linear regression of BW against test date, to obtain average daily gain (ADG). Feed conversion efficiency (FCE) was calculated as the ratio of ADG/DMI.

For effects of diet, breed and their interaction on Δ^15^N and FCE, least squares means (LSM) were estimated using a general linear model analysis (GLM, Version 9.1 for Windows, SAS Institute Inc., Cary, NC, USA). Regression analysis to determine the contribution of body composition traits (muscle and fat depth at the 10^th^ or 12/13^th^ rib, or 3^rd^ lumbar vertebrae) and ADG on the relationship between Δ^15^N and FCE were also examined using the GLM procedure of SAS, with diet and breed as fixed effects. However, breed was never significant and was therefore, removed from the model. The contribution of each variable to the model was cross validated using stepwise regression and Variance Inflation Factors (VIF) to check for multicollinearity of selected variables. Significance was declared at P < 0.05.

Preliminary GLM analysis was carried out to estimate the influence of the KEGG annotated genes, from the selected 18 steers, on Δ^15^N by fitting the significant effects (diet) as well as the relative abundance of one gene each time^17^. The 18 steers comprised of 4 CONT, 4 OIL, 4 NIT, and 6 COMB. The residuals of each model were normally distributed. We used partial least squares analysis (PLS, Version 9.1 for Windows, SAS Institute Inc., Cary, NC, USA) to identify the most significant genes associated with Δ^15^N. The PLS analysis accounts for multiple testing and the correlation between microbial genes. The model selection was based on the variable importance for projection (VIP) criterion^22^, whereby microbial genes with a VIP < 0.8 contributed little to the prediction.

## Results and Discussion

### Dietary nitrate increases isotopic N discrimination

**Figure 1:**
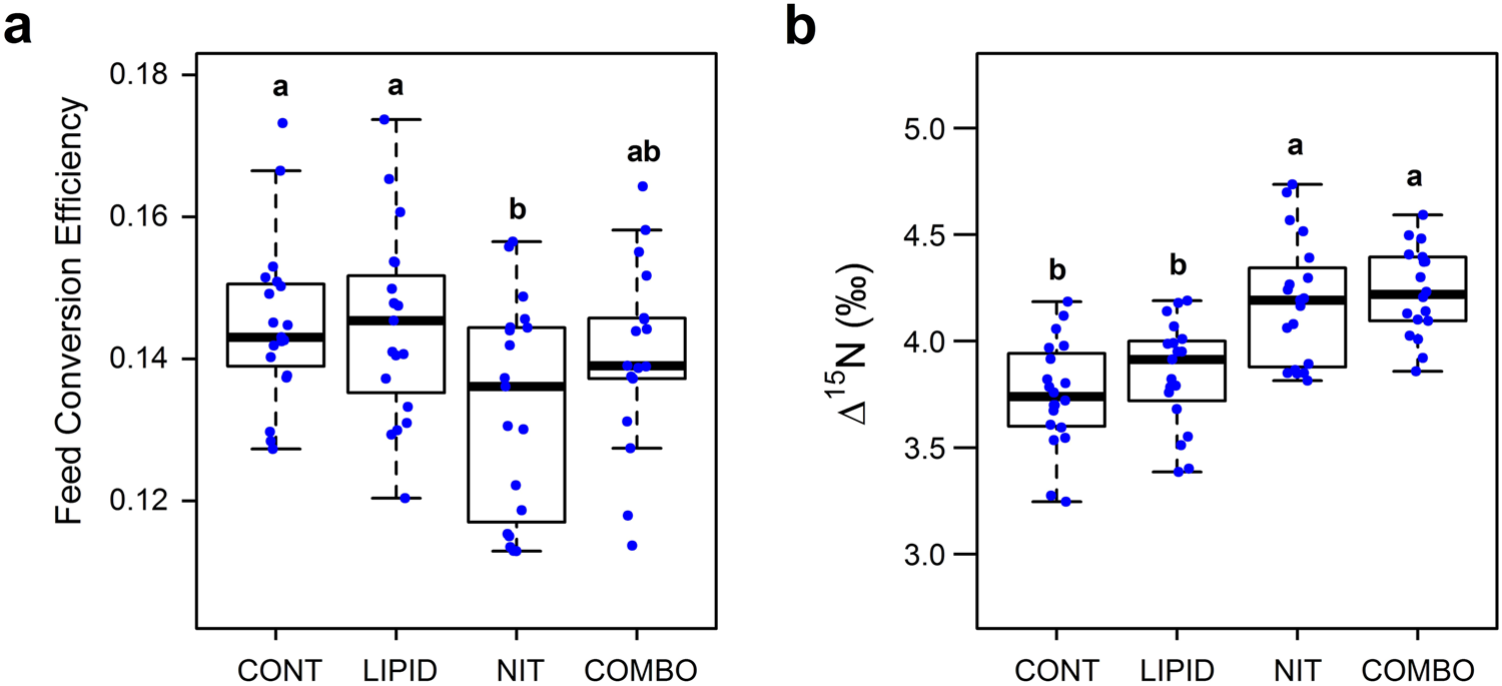
Effect of dietary additive on (**a**) feed conversion efficiency and (**b**) isotopic nitrogen discrimination in Aberdeen Angus x and Limousin x steers. ^a-b^Boxes with different superscripts have differing means (P < 0.05).

Growth and production traits of these animals have been described previously^15^. Briefly, no breed effect was observed on FCE. However, FCE was reduced (P = 0.02) in steers supplemented with NIT, compared to CONT and LIPID (Fig. 1). This reduction in feed efficiency with the NIT diet was mitigated with the inclusion of lipids to the nitrate diet (COMB).

The natural abundance of isotopic N (δ^15^N, ‰) was higher in the COMB diet, compared to NIT, LIPID or CONT diets (4.40, 4.18, 4.10 and 4.08, respectively). The δ^15^N of the NIT diet was however, numerically higher than non-nitrate containing diets. The elevated δ^15^N could be expected to reduce N isotopic discrimination in plasma proteins (Δ^15^N, ‰) of steers fed diets containing calcium nitrate, yet this was not observed here. Nitrogen isotopic discrimination was not affected by breed. This was not unexpected because animals came from rotational crosses of two breeds so that AAx animals had an expectation of 2/3 AA and 1/3 LIM genes and LIMx *vice versa*. In contrast, changing the dietary protein source altered N isotopic discrimination such that, steers fed diets containing calcium nitrate (NIT and COMB) had higher isotopic N discrimination, compared to those fed non-nitrate diets (P < 0.001; Fig. 1). Wattiaux and Reed^3^ showed that discrimination of N isotopes can occur during the incorporation of ammonia into amino acids and microbial proteins, and as dietary nitrate supplementation linearly increases rumen ammonia concentrations this may explain the increase in N isotopic discrimination. Similarly, the inclusion of dietary nitrate may increase nitrous oxide emissions^23,24^ and this may be a source of additional N isotopic discrimination.

To explore the contribution of rumen N metabolism on N isotopic discrimination we conducted metagenomics analysis on rumen content samples. We revealed 4 827 microbial genes, 1 700 of which had a relative abundance >0.01%, and we have used their relative abundances to explore possible effects of N metabolism on overall Δ^15^N. The effect of dietary nitrate is of interest in relation to possible effects of denitrification on N isotopic discrimination. Complete denitrification of NO_3_^−^ to N_2_ involves four reduction steps which are catalyzed by four main types of functional enzymes, those being nitrate reductases Nar or Nap; NO-generating nitrite reductases NirK or NirS; N_2_O generating nitric oxide reductases cNor, qNor, or qCuNor; and the two types of nitrous oxide reductases N_2_OR^25,26^. Despite deep metagenomic sequencing, we only observed two genes listed in the KEGG database, K00370 and K00371 that are part of the denitrification pathway, the first of which corresponds to the nitrate reductase, *narG*^27^. Yet, despite these two genes also being present in the dissimilatory nitrate reduction pathway, they were only observed in one animal at a relative abundance of 0.01%, suggesting that their contribution to N isotopic discrimination is likely negligible in the rumen. Investigations of denitrification genes in the rumen are scarce. The absence of denitrification genes in the rumen does not provide direct support for the suggestion that the effect of nitrate on Δ^15^N can be explained by denitrification. However, it remains possible that the KEGG representatives we mapped to, do not reflect well the rumen enzymes for denitrification and/or denitrification may be occurring in the mouth. Peterson *et al*.^24^ measured nitrous oxide emissions from cattle in respiration chambers and, based on diurnal patterns of emissions relative to methane, suggested that it may be produced by denitrifying bacteria in the mouth. Studies in dentistry literature^27^ show nitrous oxide emissions generated by oral bacteria utilising nitrate which is concentrated and recycled via saliva. The large volumes of saliva produced in ruminants, as well as the regular regurgitation and chewing of partially digested feed during rumination suggests that this process could be more important in ruminants; this would be consistent with our Δ^15^N observations, but requires verification.

Furthermore, the process of dissimilatory nitrate reduction to ammonia (DNRA) is considered to be favoured over denitrification in anaerobic, reduced environments^28^, such as the rumen. Despite the lack of evidence for discrimination during DNRA, discrimination of N isotopes may occur during the incorporation of ammonia into amino acids and microbial proteins^3^. Here we observed that unlike denitrification genes, enzymes involved in the biosynthesis of amino acids, including the small and large chain of glutamate synthase (NADPH/NADH; K00266 and K00266, respectively) were present in all animals at an average relative abundance of 0.354%. This is in agreement with previous work, which reported a low presence of denitrifying genes in the bovine rumen compared to those contributing to nitrate and nitrite ammonification (85 vs. 636 genes, respectively, out of a total 1233 involved in nitrogen metabolism)^29^. Similarly, a limited number of rumen bacterial species have been proposed as denitrifiers including *Pseudomonas aeruginosa* and species of *Propionibacterium* and *Nitrosomonas*, which have been considered transient or minor rumen colonizers in the absence of dietary nitrate^30,31^ further suggesting that denitrification is not a primary pathway of N metabolism in the bovine rumen.

**Figure 2:**
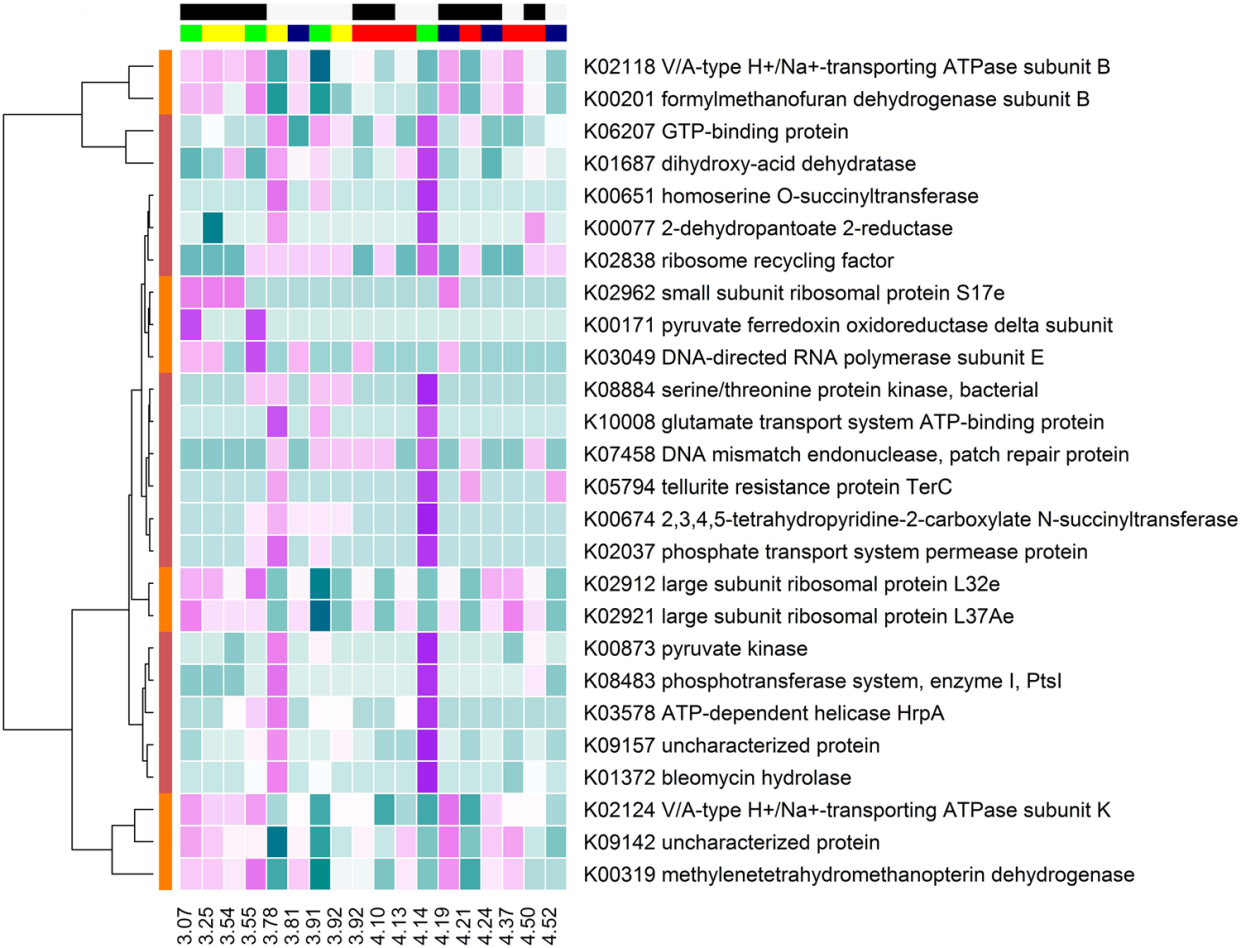
Heat map of the relative abundance of microbial genes associated with N isotopic discrimination as identified in the partial least squares analysis. The relative abundance of microbial genes (aqua = low to purple = high) changed depending on N isotopic discrimination. The coloured bars on the horizontal axis represent diet (CONT = yellow, LIPID = green, NIT = blue, COMB = red) and FCE (low = light grey, high = black). Coloured bars on the vertical axis represent the cluster in which genes were located (red = cluster 2, and orange = cluster 5). The values on the x-axis represent the corresponding Δ^15^N values for each animal arranged in ascending order.

As part of a larger study^32^, a network analysis was carried out based on the relative abundance of KEGG genes and found 15 distinct functional clusters of gene networks. To further investigate the influence of rumen microbial genes on N isotopic discrimination we performed a partial least squares analysis, firstly, on all genes whose relative abundance significantly differed with Δ^15^N. From this, 50 genes were identified belonging to either cluster 2 or 5. Subsequently, all genes from these 2 clusters (n = 195 and 92, respectively) where examined via partial least squares analysis, revealing that 26 rumen microbial genes accounted for 68.6% of variation in Δ^15^N (model variation was 67.6%; Fig. 2). Surprisingly, no genes from the Nitrogen metabolism pathway were identified as influential in the prediction of N isotopic discrimination. The identified genes have primary roles in carbohydrate metabolism, energy metabolism and amino acid metabolism. Similarly, no clear pattern of N isotopic discrimination in association with the relative abundance of genes was observed (Fig. 2) indicating that ruminal genes may have a very limited contribution to the process of N isotopic discrimination in ruminants under these conditions. It is possible, however, that gene expression is not intimately linked to gene presence, and as such, a metatranscriptomic analysis may reveal further insights into the role of rumen N metabolism on N isotopic discrimination. An important consideration is the time of sampling, as blood was sampled at d 56 of the feed efficiency test, and rumen contents were sampled at slaughter at d 154. Despite this temporal separation, the animals remained on the same experimental diets during the interim period and as reported by Cantalapiedra-Hijar *et al*.^33^, isotopic equilibrium is achieved after 45 d on an experimental diet, indicating that sampling blood at 56 d would be expected to yield similar N isotopic discrimination as if the blood samples were collected at the time of rumen sample collection.

### Relationship between Nitrogen isotopic discrimination and FCE

**Figure 3:**
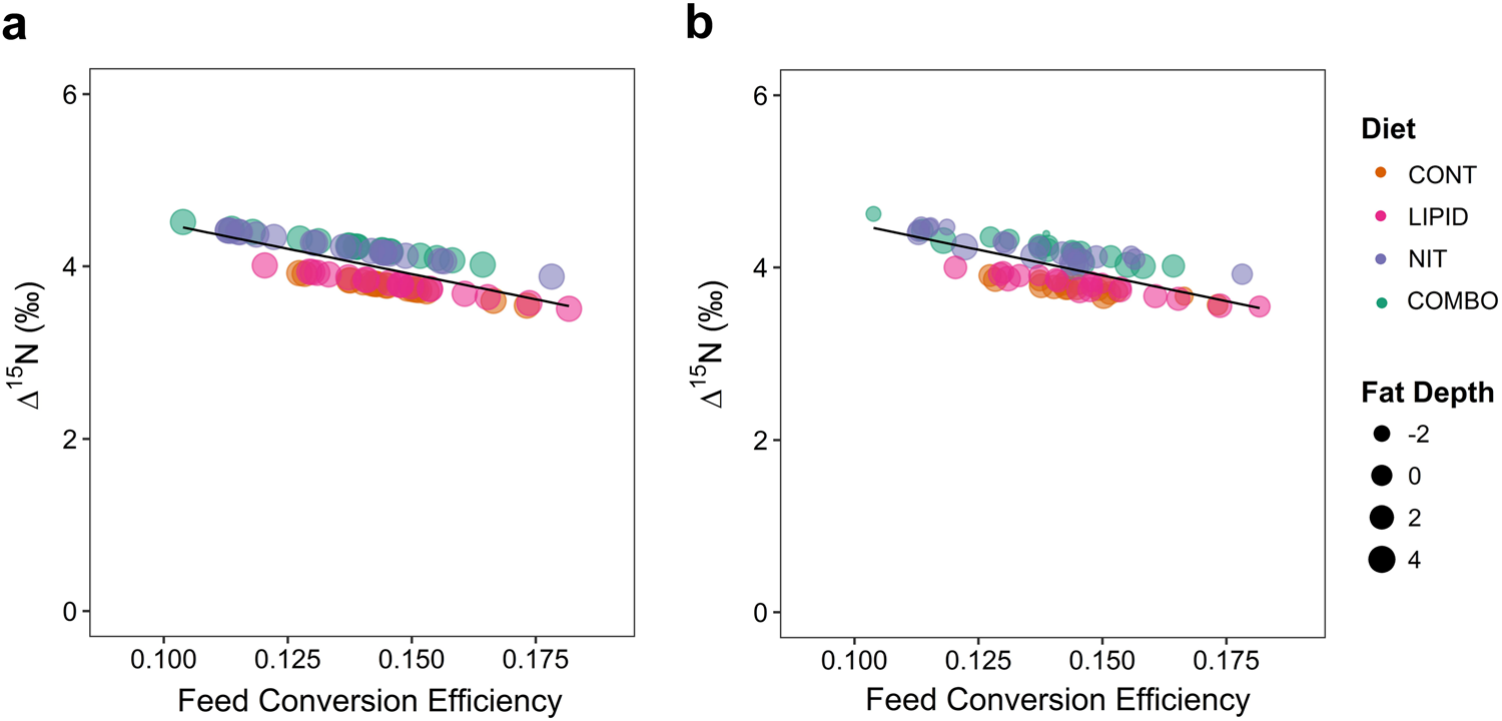
Effect of Feed Conversion Efficiency on N isotopic discrimination in plasma proteins (Δ^15^N) determined by regression analysis. The predicted relationship between Δ^15^N vs FCE including diet effect ((**a**) R^2^ = 0.534; P < 0.001) was strengthened with the inclusion of changes in fat depth at the 3^rd^ lumbar vertebrae during the feed efficiency test period ((**b**) R^2^ = 0.552; P < 0.001).

To better understand the relationship between FCE and N isotopic discrimination we conducted a regression analysis which revealed a strong negative correlation (R^2^ = 0.534; P < 0.001; Fig. 3a) between the two variables, with the inclusion of diet. This correlation was reduced to 28.7% (P < 0.001) when excluding the effect of dietary additive. The general form of this relationship is commonly observed across different species and breeds of ruminants^4,5^, indicating the potential of Δ^15^N_animal-diet_ as a biomarker for FCE. Previous work has shown that a significant proportion of N isotopic discrimination results from processes involved in N metabolism – particularly in the liver^1,2^, and rumen microbial metabolism^3^. Whilst there is a relationship between these processes and overall FCE, it seems likely that there would be a stronger relationship with the efficiency of N utilization (NUE). A few studies have linked N isotopic discrimination to NUE in sheep^34^ and dairy cattle^35,36^, but this is not so easy to accomplish in growing animals where daily N gain is difficult to measure. To explore this relationship further we included body composition parameters in our model, specifically the change in back fat depth at the 3^rd^ lumbar vertebrae over the FCE test period, which accounted for an additional (P < 0.001) 1.80% of variation in N isotopic discrimination (R^2^ = 0.552; Fig. 3b). Furthermore, we considered the overall change in body composition, i.e., the average change in fat depth across the 3 measurement sites. However, the predictive ability of the model did not increase beyond that of the single measurement at the 3^rd^ lumbar vertebrae (R^2^ = 0.556; P = 0.801). Interestingly, the inclusion of ADG over the test period, in the basal model with diet and FCE, accounted for less variation in Δ^15^N (R^2^ = 0.539; P < 0.001), compared to the inclusion of change in fat depth. The lack of contribution of ADG in the model to estimate Δ^15^N, may result from its high correlation with FCE. As when FCE was excluded as a predictive variable, ADG accounted for 43.9% of variability in Δ^15^N, including the diet effect (P < 0.001). Conversely, Δ^15^N and diet explained 30.2% (29.7% without diet) of the variation in FCE. The inclusion of both ADG and Δ^15^N explained 58.9% of variability in FCE. However, the inclusion of body composition parameters only showed significance for the change in fat depth at the 10^th^ rib, which increased the amount of variability accounted for to 60.4% (P < 0.001; Supplementary Fig. S1). Conversely, fat depth at the 12–13^th^ rib did not increase the predictive power of the model. This may be due to the short duration of the test period (i.e. the relatively small changes in measurements of muscle and back fat depth over a 56 d period). Nevertheless, our results suggest that the composition of gain, i.e., partitioning between fat and muscle may account for variation in Δ^15^N in growing steers to a greater extent than the total amount of gain (ADG). Whereas, FCE is more susceptible to changes in total gain (ADG).

## Conclusion

The small percentage of variation in Δ^15^N explained by rumen microbial genes and the lack of association with increasing Δ^15^N levels suggests that rumen microbes have very little influence on the N isotopic discrimination observed in plasma proteins of beef cattle. However, the effect of FCE on Δ^15^N was strengthened with the inclusion of body compositional changes, specifically fat accretion at the 3^rd^ lumbar vertebrae during the feed efficiency test.

## Electronic supplementary material

Supplementary Figure S1
