# Supplementary material for: Fat accretion measurements strengthen the relationship between feed conversion efficiency and Nitrogen isotopic discrimination while rumen microbial genes contribute little
Source: Sci Rep. 2018 Mar 1;8:3854. doi: 10.1038/s41598-018-22103-4 (PMC5832862; doi:10.1038/s41598-018-22103-4)
Supplement: Supplementary file 1 — Supplementary Figure S1 [file 41598_2018_22103_MOESM1_ESM.doc]

**Fat accretion measurements strengthen the relationship between feed conversion efficiency and Nitrogen isotopic discrimination while rumen microbial genes contribute little**

Sarah J. Meale, Marc D. Auffret, Diego P. Morgavi, Gonzalo Cantalapiedra-Hijar, Carol-Anne Duthie, Rainer Roehe, Richard J. Dewhurst

**Supplementary Information**


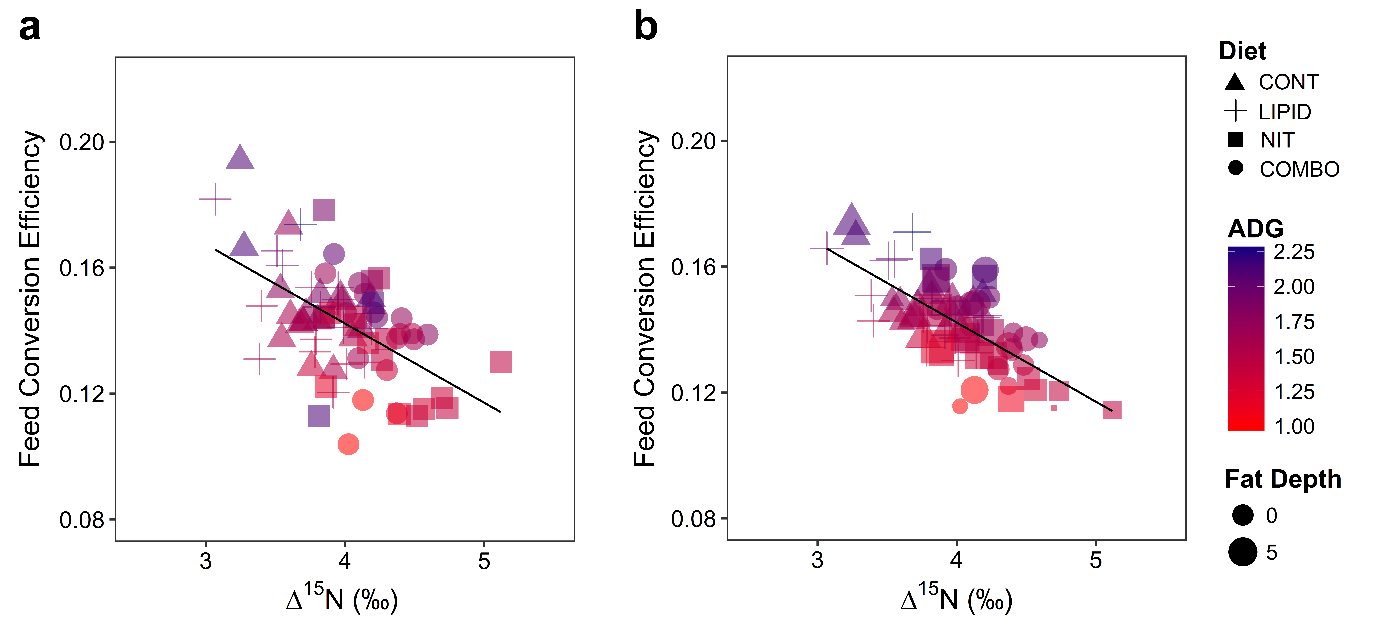


**Supplementary Figure S1. Effect of isotopic nitrogen discrimination in plasma proteins (Δ^15^N) on Feed Conversion Efficiency as determined by regression analysis.** Predicted values of FCE vs Δ^15^N including diet effect (a; R^2^=0.302; P<0.001); and including the effect of Δ^15^N, average daily gain and change in fat depth at the 10^th^ rib during the feed efficiency test period (b; R^2^=0.604; P<0.001).
